# Psychometric characteristics of the chronic Otitis media questionnaire 12 (COMQ – 12): stability of factor structure and replicability shown by the Serbian version

**DOI:** 10.1186/s12955-017-0782-x

**Published:** 2017-10-23

**Authors:** Bojana Bukurov, Nenad Arsovic, Sandra Sipetic Grujicic, Mark Haggard, Helen Spencer, Jelena Eric Marinkovic

**Affiliations:** 1 School of Medicine, University of Belgrade, Clinic for Otorhinolaryngology and Maxillofacial Surgery, Clinical Centre of Serbia, Pasterova Street 2, Belgrade, 11 000 Serbia; 2 School of Medicine, University of Belgrade, Institute for Epidemiology, Belgrade, Serbia; 3 Department of Psychology, University of Cambridge, Cambridge, UK; 4 Eurotitis Study Group, Cambridge, UK; 5 School of Medicine, University of Belgrade, Institute for Medical Statistics and Informatics, Belgrade, Serbia

**Keywords:** Otitis media, Outcome assessment, Impact, Quality of life

## Abstract

**Background:**

Recently, demand for and supply of short-form patient-reported outcome measures (PROMs) have risen throughout the world healthcare. Our contribution to meeting that demand has been translating and culturally adapting the Chronic Otitis Media Questionnaire-12 (COMQ-12) for adults into Serbian and enhancing its psychometric base on the relatively large Serbian COM caseload. Chronic otitis media can seriously affect quality of life progressively and in long-term, and it remains the major source of hearing problems in the developing world.

**Methods:**

The translated questionnaire was given twice to 60 adult patients with chronic otitis media of three types (inactive, active mucosal and active squamous disease) and to 60 healthy volunteers. Both patients and volunteers also filled the generic Short-Form 36 questionnaire (SF-36). Conventional statistical procedures were used in strategically driven development of scoring. Additionally, item responses were scaled by linear mapping against the provisional total score. Generalizability, detailed factor interpretation and supportability of scores were criteria, for the best compromise factor solution.

**Results:**

Test-retest reliability was very high (0.924 to 0.989, depending on score). The a priori content dimensions of the questionnaire were strongly supported by 3-factor exploratory and confirmatory factor analyses for content validity, separating (i) ear symptoms from (ii) hearing problems, from (iii) daily activity restriction plus healthcare uptake. The 3-factor structure was furthermore highly stable on replication. The very large effect sizes when contrasting patients with healthy volunteers, and active with inactive disease established construct validity for the total score. A strong association with disease activity and a moderate one with generic health-related quality of life (HRQoL), the SF-36, supported construct validity for two of three factors extracted (ear symptoms, and impact on daily activities plus healthcare uptake).

**Conclusions:**

Given the minimal psychometric work to date on COMQ-12, this interim sample with 120 data points adds materially to knowledge of its reliability, several forms of validity and the feasibility of profile sub-scores to supplement total scores. The good psychometric properties shown for COMQ-12 justify both its routine clinical use and acquisition of the necessarily larger sample for generality, score optimisation and the evaluation of responsiveness.

**Electronic supplementary material:**

The online version of this article (10.1186/s12955-017-0782-x) contains supplementary material, which is available to authorized users.

## Background

Adult chronic otitis media (COM) is a major health problem and still the chief cause of preventable hearing disability in the developing world [1]. It has been a relatively small problem in the developed world through the antibiotic era, but may become a larger one again if that era ends, as the growing resistance of bacteria threatens [2, 3]. The morbidity is definite and well established; due to an erosion and destruction of tissues in the middle ear. The considerable effect on quality of life (QoL) arises by two main routes: persistent infection and mal-odorous discharge from the ear, accompanied with discomfort, and loss of hearing plus occasionally, balance function. Depending on the spread of disease, its degree of activity and type, and on the appropriateness of surgical technique used, rates of ‘surgical failure’ in disease management are relatively high, especially in the active squamous form of disease, reportedly from 16 to 29.4% [4]. These patients particularly have high morbidity and resource demand, many having repeated operations. Equally, success in restoring hearing is at best modest: in over 40% of patients, hearing after surgery remains unchanged, and it can sometimes even become worse [5]. Furthermore, if initial hearing gains are accomplished, they can decline progressively over time [6].

Given the variable treatment success of surgery and hearing restoration, a comprehensive approach to the assessment of the magnitude of impact of hearing loss and accompanying symptoms in COM patients should be established. Also, the contribution of hearing problems and ear drainage considerations to treatment decisions and successful management of the disease has yet to be set out clearly. For monitoring quality of healthcare and targeting or revising treatment strategies in COM, good measurement of wider impacts as well as physical health status and hearing are needed.

### Measurement challenge

In recent years, even rare conditions have seen a flood of short-form instruments (questionnaires) developed, reported and applied for routine applications as patient-reported outcome measures (PROMs) [7]. There have been several such attempts to assess QoL in adult COM, and to find validating relationships between this and hearing levels (HL, audiograms) or other traditional clinical measures (examination data, recurrence rates and number of repeated office visits). Existing COM questionnaire measures seem not to well represent the perceived hearing disability, unless using many hearing items (so incompatible with short-form PROM status); and they do not correlate well with overall quality of life [5, 8]. Furthermore, measured hearing disability and QoL seem to relate poorly in adult COM to the presumed bases in HL and other clinical data. Within the developed world, these patients are mostly of lower socioeconomic status and lower education, possibly limiting sophisticated measurement, and the disease impact might synergise with such factors; both these considerations would demand many items to achieve measurement precision, also large sample size. The social domain of QoL in COM patients has never been assessed very fully, despite these patients experiencing social isolation due to both the hearing disability and the disease symptoms. A good general instrument should therefore reflect this also.

The present article reports Phase 1 of a 2-phase psychometric development project building on a review of the existing Chronic Ear Survey (CES) [9] and Chronic Otitis Media Outcome Test 15 (COMOT-15) [10]. The rationale of Phillips et al. [11] in composing the successor, the COMQ-12, was to use prior good items for hearing and ear symptoms and to succinctly reflect wider impact; the resulting COMQ-12 showed sufficient preliminary construct validity to justify further development and evaluation. The present work uses the Serbian caseload (relatively high for a European country) to take the development forward. Reference data now exist also on Flemish (Dutch) and Russian versions of COMQ-12 with further languages pending, but minimal further psychometric or other evaluation has been reported [12, 13].

To help COMQ-12 take its justified next steps, we have obtained a COMQ-12 translation and a substantial reference sample for Serbian to undertake more detailed psychometrics. The Phase 1 study reported here documents (a) the capabilities and potential of the instrument more fully than has been done so far, addressing three forms of validity (content, construct and metric), plus reliability (test-re-test) and (b) the potential for profile sub-scores. For such a very short instrument, the heterogeneity of disease facets involved needs to be bridged to provide some total severity score; that aim is appropriate, but achieving it with few items may threaten the viability of sub-scores for profiling. This conflict of aims reflects (and may be exacerbated by) an ambiguity over the a priori affiliation of two COMQ-12 items that are “wild cards” among COM symptoms: tinnitus (associated with hearing losses of both middle and inner ear origin) and dizziness (an inner ear complication, but also a central age-related symptom correlate). Both these symptoms have large, though variable, effects on HRQoL [14], justifying inclusion a priori in any ear condition. To pursue aim (b) we address two related strategic measurement issues, both practical, although the 1st also has theoretical content: (i) is there a good factor structure which can directly support content validity and probably show construct validity? Then, if so, (ii) can the symptom items originally included for totaling purposes also contribute in a sufficiently structured way to support worthwhile sub-score profiling with COMQ-12? Alternatively, aim (ii) might only be achievable by adding further items to fill revealed gaps; so, decision on (ii) is urgent, to avoid possibly premature standardization preventing achievement of that aim.

## Methods

### Translation

Linguistic and cultural adaptation of questionnaires should follow well established principles and procedures. After permission from the British originators, COMQ-12 underwent forward translation into Serbian by a medical translation agency using two independent translators (Serbs fluent in English). Small differences between the two translated versions were discussed and reconciled by the agency editor (a native Serbian speaker with formal education in Serbian language, also fluent in English). This stage captured two instances of translation error with missing content, solved by minor changes in the reconciled version to ensure conceptual equivalence. After a native English speaker had back-translated the reconciled version into English, differences from the original English were discussed by one of the originators with the 1st author for corrections and suggestions (only one back translation error of lexical type found and corrected). Piloting of the resulting preliminary Serbian version with debriefing on 10 consecutive patients showed that no modifications were needed for the final version of the questionnaire (see Additional file 1).

### Participants and data

Consecutive patients diagnosed with chronic otitis media at the Clinic for Otorhinolaryngology and Maxillofacial Surgery, in Clinical Centre of Serbia, Belgrade in the period October 2014 to mid-January 2015, were included. These (60) were supplemented by 60 healthy volunteers. This Phase 1 study sample was heterogeneous, comprising three roughly equal subgroups of patients with different stages and activity of their disease: 20 patients had inactive chronic otitis media, 21 had active mucosal disease and 19 had cholesteatoma. The questionnaire was self-reported, and all patients were asked to fill one copy of COMQ-12 twice, at first visit, and again 4 weeks later. There were no missing questionnaires or single items in this sample. They also filled in the Serbian version of the generic health status measure SF-36 [15] on the first occasion only. Supplementary patient data collected included age, gender, and pure-tone audiograms (both ears) by air- and bone- conduction hearing levels (contralaterally masked if the obtained interaural difference for the frequency being tested was greater than 20 dB, and always masked when determining bone conduction thresholds), plus in the clinical group, the history of disease and oto-microscopic findings. The normative reference volunteer group was a comparable convenience sample without previous history of middle ear disease, composed from hospital staff members of various grades. The larger sample for Phase 2 has patients only, including those from Phase 1, and post-intervention longitudinal follow-up.

### Statistical strategy and techniques

The main aim was to use the 120 data-points to make well justified interim decisions about the scoring of the instrument in basic respects (item contributions, supportable dimensions) and to specify issues remaining for an optimal version, which might be addressed on a larger sample and with longer-term follow-up. For the main standard statistical procedures (eg chi-squared, t-tests, (M)ANOVA and exploratory factor analysis) we used the satisfactory versions in SPSS version 22 and 23. The typical preliminary approach ‘significance of any effect’ (gambling over existence of effects in small samples) is not scientifically appropriate, although we do make appropriate use of *p*-values for determining inclusion of model terms. Given the need for wider appreciation of the dual impact of COM, plus the apparently modest and variable therapeutic gains from surgery it is important to show and discuss effect sizes (ESs) somewhat precisely [16]. The most widely used ES measure, and best for appreciating differences in continuous data on unfamiliar scales, is the standard deviation (SD) effect size [17], for which near-normal distributions are desirable. The normality of raw descriptives and of model residuals was inspected visually and quantified by skew and kurtosis indices (with standard-error-based tests), supplemented by the Kolmogorov-Smirnov statistic. For documenting content validity, exploratory principal component and factor analyses (EFA) were performed with Varimax orthogonal rotation, separately for baseline and re-test occasions. After examining stability of factor structure, we combined the 2-visit data at the item level, to maximise reliability of decision on the optimum basis for extracting factor scores. Confirmatory factor analyses (CFAs) to assist decisions about cross-loading were run via structural equation modelling (SEM) [18] in the SPSS adjunct package AMOS. To appraise construct validity, these CFAs were then extended to full SEMs by adding a priori links from other relevant patient characteristics available; in this CFA we also altered two of the 3 (necessarily low) factor inter-correlations to regressions, allowing both hearing and ear symptom factors to predict wider impact.

## Results

### Descriptive preliminaries and known-groups construct validity

Table 1 details demographic and clinical characteristic of patients and unaffected individuals (‘controls’), also baseline severities in a limited range of variables at the first visit: air conduction thresholds for better- and worse-hearing ears, plus the raw (ie unscaled and unweighted) COMQ-12 total. The chief mean HL difference in the baseline data, between the two active groups combined and inactive, emerges as 20.687 on the affected ear, and is almost identical at 20.156 on the worse. The SD effect size for this affected ear mean HL difference against pooled group absolute variability is very large at 1.56 SD (*t* = 5.685; *p* < 0.001). There was no reliable difference between study and control group in the mean age (t-test: *t* = 0.446, df = 118; *p* = 0.657) nor in the gender balance (Chi-squared = 0.137, df = 1; *p* = 0.711). Similarly, there were no such differences between active and inactive disease (respectively t-test: *t* = 0.667, df = 58, *p* = 0.507; Chi-Sq = 0.543, df = 1; *p* = 0.461). Gender was therefore not used further, and age failed to condition any of the later comparisons of interest (but see later figure footnote).

**Table 1:** Basic 1st-visit descriptives of clinical groups and healthy individuals in Phase 1 Study

Data at 1st Visit	Unaffected controls *N* = 60	Inactive COM *N* = 20	Active mucosal COM *N* = 21	Active squamous COM *N* = 19
	Mean (SD), Skew	Mean (SD), Skew	Mean (SD), Skew	Mean (SD), Skew
Age	43.52 (16.03), 0.153	44.20 (15.85), −0.180	45.47 (16.89), −0.173	36.47 (15.59), 0.891
Better ear average HL		24.81 (11.83), 0.885	32.14 (16.74), 1.352	33.09 (21.46), 1.393
Worse ear average HL		38.89 (11.76), 0.745	59.76 (17.26), 2.255	59.34 (16.32), 0.698
Raw total COMQ-12	1.02 (1.88), 2.345	20.10 (8.40), 0.000	30.63 (9.99), −0.843	29.68 (11.75), −0.055
Disease duration %-ile split		50.0,50.0	38.1, 61.9	36.8, 63.2
Educational level %-ile split	55.0,45.0,0	50.0, 45.0, 5.0	42.9, 52.4, 4.8	26.3,68.4, 5.3

The percentages male and female in the COM groups totaling 60 were 43.3 & 56.7% and the percentage with left side affected (ie candidate ear for operation) was 51.7%. The reported disease duration is expressed as the near-median split at 8 years, although it is recognised this figure may not be veridical. Educational level is dichotomised at primary plus secondary, versus beyond secondary, then missing data. The apparently greater educational qualification level of the squamous relative to other groups is not significant (Fisher Exact p, 2-tail 0.16). In each variable, skew standard errors are 0.512, 0.501, 0.524 for the three clinical groups and 0.309 for controls. In the main analyses later using HL, the value is taken from the affected ear, ie the candidate for operation, as having more disease-relevant variance. This is also in nearly all cases the worse ear. Though differing little here, age was extensively explored as a control covariate in analyses subsequently reported, but it never improved model fit

Average total raw score in the clinical group was 26.82 (SD 11.0) out of a maximum score of 60, ranging from 5 to 46, with an acceptably near-to-normal distribution (Kolmogirov-Smirnov statistic 0.097, p~0.200; Shapiro-Wilks statistic 0.967; *p* = 0.098). The three patient groups have tractably slight skew and homogeneous SDs around 10.00, so providing useful gradation in the clinical population. The 60 healthy controls have extreme positive skew (floor effect with few non-zero responses). This entails that their very narrow SD cannot provide the normal reference distribution nor even meaningfully be incorporated into a pooled variance in the way common for computing the SD effect size. Using the square root of the pooled variance estimate from the clinical groups only, the health controls’ mean total raw score was far (approximately two SDs) below even that of the inactive group (Z = 6.997; *p* < 0.001) and about 3 SDs below the two active groups combined (Z = 8.689; p < 0.001). Thus, the questionnaire correctly locates the patients in a distinctively pathological part of severity space. As a more relevant demonstration, although the two active groups do not differ from each other, they do differ jointly from the inactive group. In the language of ‘significance’, this disease activity effect is reliable on the present sample size (*t* = 3.67; df = 58; *p* = 0.001), but more importantly has large enough effect size (1.005 SD) to conclude that known-groups construct validity for the ear symptoms score is shown, and to proceed to determine and use the factor structure.

### Internal consistency and test re-test reliability for total

These checks were performed on unscaled items to justify work towards item scaling and weighting. Cronbach’s alpha coefficient, the conventional index of consistency or factorial purity, aggregates the inter-item correlations to reflect a mixture of underlying reliability and validity. For first visit this was 0.821, well above the conventional threshold of acceptability (0.7) [19]. The Cronbach’s alpha at Visit 2 retest was slightly better (0.84) possibly due to increased participant familiarity with questionnaires. The between-visit intra-class correlation coefficient (ICC) value for the total was very high at 0.985 (95% confidence interval: 0.975 to 0.991). Further reliability data (for scaled and factor scores) are given later.

### Exploratory factor analysis and item scaling

We examined further inter-item relationships and dimension structure of the unscaled items with principal component and factor analyses. All 12 questions showed sufficient loadings on the first principal component (1st PC) to be retained and scored in the total (minimally loading item for the two visits, tinnitus: 0.429, 0.419, requiring further scrutiny in terms of profile contribution). Henceforth, we use the saved weighted 1st PC values as giving a more optimised precise metric for total score, but this is identical in concept to the previously mentioned raw total and correlates with it highly, even at first visit (*r* = 0.997). The 1st PC also offers the most reliable basis for later optimising the items’ contributions to the 1st PC total itself, by scaling the intervals between their response levels in terms of units on that 1st PC score.

For scaling these intervals, we heeded two possible limitations: (a) in the scale-determining variable to be predicted by item regressions, the presence of only about 4 high-loading items per factor make it prudent only to use the 12-item principal component as scaling criterion at this stage, for reliability’s sake; and (b) in the independent variables (ie the response levels to be given coefficients) some categories have few instances (so wide standard error) to reliably characterise extreme response levels for their association with the generally extreme total. We have therefore deferred to the larger Phase 2 the final optimised scaling of items by their various levels’ contribution to respective factor scores on which items load highly, rather than to 1st PC. Following the method previously described [20], and with the unscaled 1st PC total for the 2nd visit’s data initially as the preliminary dependent variable, we ran all 12 item-scaling models using the univariate ANOVA version of the General Linear Model. This gave 12 sets of up to 6 decimal scaled coefficient values with ‘missing’ and ‘not sure’ included; these represent the mean differences in the preliminary total values produced by those respondents who gave the particular integer response level to the particular item. There followed several adaptive stages of scaling, iterative in nature, to diminish any influence from the starting point, although from previous large-sample experience, one iteration generally suffices because of convergence. For the second round, the to-be-predicted preliminary total was the scaled output from the first round (visit 2), applied to the visits separately. The high agreement obtained between the scale values for the two visits’ data then justified averaging their scaled values for each response level for items to total and provide the dependent variable in the next round, but items were again scaled for each visit separately. We finally averaged scaling coefficients for Visit 1 and 2 data to get reliable general scaled values from which we then defined a principle component weighted total (Table 2 and the further comments on scaling in Additional file 2). This convergence-based single scoring system was then imposed on the separate visit data for purposes of factor structure choice and definition of PC total score (Additional file 2 and Table 3); for reliability, further analyses proceeded on the 2-visit averaged data with this average-derived scoring.

**Table 2:** Provisional scaled item response values

Response Level	Q1	Q2	Q3	Q4	Q5	Q6	Q7	Q8	Q9	Q10	Q11	Q12
0	0.000	0.000	0.000	0.000	0.000	0.000	0.000	0.000	0.000	0.000	0.000	0.000
1	0.582	0.021	0.462	0.000	0.000	0.449	0.000	0.704	0.000	0.422	0.733	0.698
2	1.493	0.021	0.880	0.745	0.653	1.091	0.000	0.704	1.423	1.072	1.099	0.852
3	1.493	0.206	0.880	0.745	1.187	1.268	1.009	1.492	1.423	1.727	1.632	1.695
4	2.079	1.143	2.199	1.421	1.720	1.509	1.446	1.657	1.423	1.727	1.710	2.141
5	2.354	1.356	2.333	2.324	1.890	2.302	1.206	1.749	1.423	2.110	1.926	2.789

**Short Item Key:** 1 Q1 - Draining ear; Q2 - Smelly ear; Q3 - Hearing at home; Q4 - Hearing in noise; Q5 - Discomfort/pain; Q6 – Dizziness; Q7 – Tinnitus; Q8 - Activity restriction; Q9 – Need to limit exposure to water; Q10 - GP visits; Q11 - Taking medicines; Q12 - Overall QoL impact of hearing problems

Entries are the adopted item response values for the 5 levels for each of the 12 questions when regressing them against a preliminary total score. Here that dependent variable was the total 1st PC total (out of 5 X 12 = 60, unscaled, but scored continuously as integers), for the average of 1st two visits so in effect a grain of 1/120 in the dependent variable. The exemplified independent variable category means estimated are the set from visit. The 12 scaling regressions shown in Table 2 are done individually for the chosen dataset eg Visit 1, 2 combined), taking the responses of 0–5 as category labels for their means in the raw total to be estimated from data by regression mapping, not as integer arithmetical values

**Table 3:** Percentage of variance explained by the factor solutions in Additional file 2

V1 3-FAC			V2 3-FAC			V12 average 3-FAC			V12 average 4-FAC		
Rotation SS Loadings			Rotation SS Loadings			Rotation SS Loadings			Rotation SS Loadings		
EV	% variance	Cum %var	EV	% variance	Cum %var	EV	% variance	Cum %var	EV	% variance	Cum %var
2.818	23.484	23.484	3.152	26.267	26.267	3.008	25.065	25.065	2.626	21.884	21.884
2.466	20.550	44.033	2.436	20.297	46.563	2.520	20.998	46.064	2.167	18.060	39.943
2.282	19.021	63.054	2.055	17.126	63.689	2.182	18.186	64.249	2.083	17.362	57.305
									1.744	14.531	71.836

EV = post-rotation eigenvalues; these are typically more evenly spread in their values than before rotation, Cum %var. = cumulative percentage of the variance explained by factor scores, ie by adding the current absolute percent variance explained to the previous

The first two fields show straightforward 3-factor solutions on the data from each visit separately but with scale values based on Visit 1 and 2 averaged data combined, making the scaling identical across all 4 fields although the data sources differ. In the last two fields, the item data themselves are averaged for the two visits, so both the 3- and 4-factor solutions proceed on this same averaged Visit 1 & 2 data. The main text, supported by Additional file 2 b explains why visit-averaged data and then the 3-factor solution are preferred

The value of scaling is illustrated by the present clear falsification of the assumption that is made when just using raw numerical ratings: that the intervals between integer response levels are equal. One example, is whether the supposed difference of 1.0 between ‘4’ and ‘5’ is truly equal in distinguishing overall severity to any other such unit interval eg the 1.0 between ‘1’ and ‘2’. The scaled values show that for three-quarters of all items (exceptions 4, 6 and 12 being acceptable), this assumption is seriously false. Thus, scaling can correct for the evident ceiling effect in 9 of the 12 items, allowing each item to do more work where it can. The note to Table 2 and supplementary information in Additional file 2 gives further examples.

We next compared parallel factor analyses on unscaled versus scaled versions of the items to explore the appropriate factor solution. In the sequence of such strategic decisions, this comparison was run in the first iteration on the 2nd visit data, as participants became more familiar with the questionnaire format, so give data slightly less variable in a general sense. For unscaled item response levels, 3 factors had explained 63.25% of the variance at visit 1; for scaled item response levels, this had been no higher at visit 2 (62.90%), but the 3rd rotated eigenvalue rose from 1.766 to 1.854. On the basis that scaling may serve partly to aid the weaker factors, and that reliability was also no worse with scaled data, this favoured adopting scaled data for determining the best factor solution via subsequent factor analyses. For unscaled items, there had been four factors with eigenvalue > 1.00, but for scaled items there were only 3 at this second visit. Because false-positive errors are possible in factor identification, “finding” more factors does not necessarily make a solution better. The parsimony of factor solution for various applications is also a goal; 3 factors are more supportable on 12 items than are 4.

The final model-choice approach to deciding between a 3- and a 4-factor solution (on item-scaled and visit-averaged data) is set out in detail in Additional file 2 and Table 3. This was done based on the visit-averaged data as justified in the first part of Additional file 2’s table. There are four main reasons to not generally favour the 4-factor solution on the basis of these data: (a) the amount of cross-loading is greater with 4 than with 3 factors, giving less neat factor interpretation, (b) the minimal structural criterion of having 3 distinct high-loading items is failed for one of the 4 factors; (c) there is minimal gain in the performance indices in Table 3; and (d) the last rotated eigenvalue is respectably high, around 2.00 with three factors, but less so with four (Table 3). The conventional narrow rule using all and only the number of unrotated PC eigenvalues above 1.00 would also be failed here (the 4th was 0.910); however, for eigenvalues between about 0.90 and 1.30, the four considerations (a)-(d) above are also needed for a good decision.

Thus, we proceed with three factors: ear problems, hearing problems and combined impacts on daily activities and healthcare uptake. Multiple analysis of variance showed no difference in any factor between the two active forms (minimum pairwise *p* = 0.597), so this group variable was collapsed to active/inactive.

### Comprehensive view of construct validity including confirmatory factor analysis and path model

**Fig. 1:**
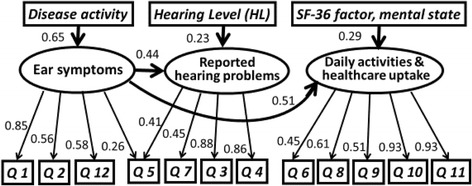
Combined Confirmatory Factor Analysis and cascaded path regression model implemented in SEM. The graphical convention for expressing structural equation models has observed variables as rectangles and underlying latent variables (essentially, factors) as ellipses with loading onto marker variables (such as COMQ-12 question items here) as an outwards arrow. For additional simplicity and clarity here: **a** loading arrows are made thinner than those for substantive regressions, **b** observable variables determining construct validity are italicised, but observable question items are not, and **c** the error terms for the observable variables usually shown as a circle plus arrow into the for each are omitted. The juxtaposed numerals are standardised regression weights (SRWs) a universal metric and one type of effect size, similar in concept to the factor loadings in Additional file 2, but on a different scale. No ordinary item loading has standardised regression weight SRW less than 0.45, except the two for the cross-loading item 5 (discomfort/pain) at 0.41 and 0.26, so all items are doing useful work ‘marking’ the underlying factors (latent variables -- ellipses). One marginal link had remained in the first two CFA models, the inter-factor correlation between hearing symptoms and generic impact. However, in the third CFA, and in the full SEM this is omitted as not necessary (CFA) and not improving fit (SEM). Major contrasts in link strength are seen within the construct validity links. The large SRW for disease activity influence on ear symptoms score factor (0.65; p < <0.001) and the moderate one for SF-36 on activities and uptake (−0.29; *p* = 0.013) contrast with the small and marginal one for hearing level on hearing factor score (0.234; *p* = 0.066)

The ‘significance’ of some expected correlation or difference does not well quantify validity. We therefore performed confirmatory factor analysis (CFA) on the combined visit data via structural equation modelling [18] (SEM) using SPSS-AMOS. The CFA in the lower half of Fig. 1 expresses the structure in a graphic and comprehensible, but formally testable near-equivalent of the 3-factor solution in EFA.

The latent variables in CFA are broadly but not precisely equivalent to EFA factors, as all low loadings are forced to zero unless explicitly recognised by dual linkage. In EFA the Varimax rotation uses the signs and magnitudes of the lower-loading items to enforce zero inter-factor correlation; in CFA contrastingly, at least k-1 inter-factor correlations have to be allowed and estimated in a k-factor CFA. The regressions between latent variables in SEM express these as potentially causal influences. The rows in the associated Table 4 summarise the trade between goodness of fit and parsimony, as the four strongest cross-loadings are reduced first to two, then to just the one significant one (See Figure legend for further detail). This was done in decreasing order of magnitude of cross-loadings (as suggested by the 3-factor EFA in Additional file 2) by backwards elimination. Also, deleted in the third CFA was the weak and unnecessary factor link between generic impact and the hearing factor. This left the comparator model for the next step. We then turned the CFA model into a full path (structural equation) model by adding (in the upper half of the figure) three a priori construct-validity links (a) disease activity influencing ear symptoms, (b) SF-36 psychological health, influencing responses about the daily activities and healthcare uptake, and (c) measured HL influencing reported hearing problems (weak, *p* = 0.066, but note that *p*-values provided by AMOS software are susceptible to which link is specified as reference, so it is more useful to apply a general notion of marginality to SRWs below about 0.28 with this sample size). A set of links such as a, b, and c is often called the ‘conceptual model’, with the factor analysis being called the ‘measurement model’ and their mutual constraint enhances validity.

**Table 4:** Parsimony-adjusted goodness of fit for 4 SEM models of CFA and causal cascade pathways

	Chi-sq	df	RMSEA	AIC	N better models found in 300 k permutations
CFA1	59.934	47	0.068	145.934	88
CFA2	64.037	49	0.072	146.037	24
CFA3	78.008	51	0.095	156.008	11
SEM	182.394	87	0.136	278.394	2

This table reflects the deletion of marginally loading items, and the addition of variables and links for one SEM (as in the Figure) to supplementing the CFA with construct validity links from disease activity, HL, and SF-36. Significance of chi-squared is uninformative (as data always differ highly significantly from a model’s predictions), but chi-squared values are the basis of calculating other indices of model fit. Lower df for CFA1 means fewer degrees of freedom in the residual error, as more df are being ‘spent’ on links for the sake of goodness of fit (GoF), making a more complicated, but less parsimonious model (CFA1&2 compared to CFA3). The RMSEA (root mean square error approximation) is the most generally used index [18] of GoF. The lowest (best) value here of RMSEA, 0.068, represents good fit, verging on excellent (criterion RMSEA <0.05) and all loading links are very highly significant except the weaker cross-loadings (candidates for elimination). This is for the first CFA with four items (Qs 5, 6, 7 and 9) cross-loading on a second factor, plus all three of the inter-factor correlation links realised. These links are next reduced to two (CFA2), and then to one (CFA3, this also omitting the non-significant link between the hearing and activities/uptake factors). As determined by the quality of the EFA solution, all these CFAs are highly parsimonious, with the parsimony-weighted Akaike Information Criterion undercutting the reference ‘saturated’ value of 180. The SEM (last row) adding the three presumably causal links from disease activity, HL and the SF-36 mental well-being factor re-derived for this sample comes close to absolute parsimony (Akaike AIC 278.394 only slightly higher than the target saturated value of 270) but with cross-loading of Question 5 on ear problems marginal at *p* = 0.085

Table 4 shows that the absolute fit of this path model was less good at 0.136 than the conventional criterion (RMSEA preferably ≤0.10 for ‘good’), and it was also poorer than the fit seen for the pure CFAs (For details, see Figure footnotes). This is because high general association (alpha) among comparable questionnaire items that are visibly consistent is relatively easy to achieve, despite here pushing the limit of a small number of high-loading items per scorable factor (only 3 or 4), making the grain of the data coarse. Also, as expected from the literature, only one of the three construct validity links (observed disease activity➔ear problems) is strong; in contrast, all but one of the factor-loading links are strong, reflecting the consistency between intrinsically similar questionnaire items. However, the permutation index tells a complementary and favourable story of structural uniqueness, whereby the model grounding in non-questionnaire data can be judged good, despite limitations in the data. As the number of CFA links is reduced by eliminating the weaker ones, there remain fewer (although still thousands of) discoverable models of equivalent structure, and of these fewer can have comparably good fit. On transitioning to the path SEM, the number of possible models of similar structure expands again, combinatorically; but most possibilities are not sensible and so most have relatively bad fit. Thus, only a negligible number of equivalently- or better-fitting models than the present SEM are found, putting the SEM path model structurally in a good light. Of 300,000 random permutations of the variables around the model, only two were better than the path model shown. Thus, the anchoring framework from the CFA and SEM, goes far beyond the conventional mere inter-correlation of within-score items (Cronbach’s alpha); the directions of the 2 inter-factor links and the 2 significant construct validity links are causally plausible, so give the COMQ-12 a causal and theoretical as well as empirical underpinning.

### Reliability with principal component and factor scoring

Table 5 gives the now scaled test-re-test reliability indices for the weighted 1st principal component total and for the now justified factor scores, as saved from the 3-factor EFA solution. The scoring bridges any visit difference efficiently, by imposing the preferred combined-visit derivation formula as single basis of scoring on the separate-visit data. Whilst reliability in the sense of precision of measurement is inevitably limited with 3 or 4 items, reliability in the sense of test-re-test stability of responses in these patients is evidently high.

**Table 5:** Test-re-test reliability of total and factor scores from EFA before and after scaling

Item scaling & scoring	Raw Total, Unscaled	1st PC Total, Scaled	Scaled Fac1 Impact^a^	Scaled Fac2 Hearing	Scaled Fac3 Ear symptoms
Scaled and scored as factors	0.989	0.983	0.968	0.924	0.944
Scaled, but scored as discrete item sets using high-loading items only	–	–	0.985	0.932	0.963

All reliabilities exceed the requirement for practical application, even those of the factors. Wider implications of reliability are addressed in the Discussion

^a^ Daily activities and healthcare uptake; name abbreviated for formatting

## Discussion

### Reliability of total scores in COMQ-12

Promising basic properties of the item set were previously documented on an English population [11], but this is the first detailed psychometric analysis of COMQ-12 with over 100 data points. Given the care over translation, and the well-defined condition (so circumscribed patient population, unlikely to be highly culture-dependent), it is not surprising that good reliability and consistency are shown for COMQ-12 total score in the Serbian version. To justify assessing individuals, scales should be fairly homogeneous (of which one index is the ability to extract only one convincing PC). For application, Cronbach’s alpha (essentially a homogeneity index) was traditionally required to be above 0.8 (good), or even 0.9 (excellent) [19]. However there are strong psychometric counterarguments that very high alpha-values, especially if used as the only development criterion, can breed superficial or overly narrow measures [21]. They may also be unrealistic for the total of a short-form outcome measure because in work on quality of life the generic aim essentially guarantees heterogeneity. Here, in order to reflect the three domains necessary and provide some at least semi-generic information on disease impact, the item set has also to be heterogeneous, creating a conflict of aim where profile sub-scoring is desired. The 3-factor solution requires homogeneity within factors, but this makes the total item set inhomogeneous across factors, so the overall alpha values for total score of 0.825 and 0.847 (visits 1 & 2) can be taken as assurance that the total refers to a sufficiently coherent construct to be used in clinical settings. Similar values for factor scores reflect the gain from within-factor homogeneity, offsetting the loss of reliability with fewer high-loading items. Of previously reported alpha values for COMQ-12, one was slightly higher than ours, 0.889 for the English version [11]. For the Dutch version, the ICC value was 0.859 [12], comparable with the present one, suggesting that the English sample may have less heterogeneity and so less clear ability to support a factor structure.

### Item loading pattern and satisfactoriness of factor solution

On scaling item response levels, the number of EFA eigenvalues >1.0 dropped to 3, but giving an increase in the total variance explained. This comparison and the stability seen between the Visit 1 and replicate Visit 2 factor solutions suggested that reliability and generalizability would be favoured by adopting scaled and averaged data both for score definition and for addressing validity issues. These three pre-requisite decisions taken, we justified extracting 3 factors in rotated Varimax EFA; the similar percentages of total variance explained (25% for daily activities and healthcare uptake, 21% for hearing, and 18% for ear symptoms) stem partly from the equal numbers of highly loading items. This supportability of 3-factor profiling combines with interpretative labels reflecting the intended content domains, rooted in the traditional clinical appreciation of the symptomatology, to give a satisfactory solution.

Cross-loading is not a major problem in this sample; unsurprisingly, CFA showed it in the 2-aspect ‘hearing problems get you down’ (Q12: QoL in Table 2), although the example remaining in the full grounded SEM, for pain discomfort (Q5) is less explicable. Wild card items were not an obstacle, with tinnitus affiliating mainly with ‘hearing function’ and dizziness with ‘ear problems’. The degree of cross-loading seen is acceptable, and in a very short instrument mainly for PROM applications, where the total score on small samples will probably be most used, some cross-loading may even be helpful. This reality, with the near equal division of the high loadings into four or five items for each factor, discourages any decision to add other items that would also create a need to re-standardise. COMQ-12 is shorter than many other such instruments, yet offers both totalling and a supportable set of sub-scores. The fundamental psychometric analyses in our work have been justified to achieve some certainty about this conclusion and proceed next to quantitative optimisation.

### Forms of validity

The metric validity was slightly improved by item scaling, suggested by its achieving a more satisfactory factor solution. This still requires further investigation on a larger sample giving more instances of rare extreme response levels, to quantify the role of such scaling in optimising forms of validity including responsiveness of the COMQ-12 to change. The present case/control effect size was very high, and large effects were also previously reported [12, 22] as expected for a disease-specific questionnaire, a pre-requisite for considering other forms of validity. More importantly, since the questionnaire aims to assess patients with the active form of COM, the large effect size using the 1st PC total score for disease forms active/inactive over 1.00 SD showed high and relevant construct validity (in this example it could also be called by the sub-type names of ‘discriminant’ or ‘known-groups’ validity).

The expected regression between HL and the hearing score is only weak. Among possible explanations are insufficient items to bridge the gap between an objective measure of threshold sensitivity and a reported measure having many determinants other than HL (as shown in children [20]). Notably, the major effect on hearing in COM is in the worse ear. However, in Table 1 the degree of variation on the better ear is comparable, but it is only correlated moderately with HL in the worse (*r* = 0.557). Classically the major contribution to auditory disability and to experienced problems is from the better ear [23], which here may be minimally related to the COM, leading to some dissociation between hearing measurements and item responses about this condition of chief concern. The chief issue here is practical: whether there is a justification to add more hearing items to make a COMQ-13 or −14? The dissociation suggests that the aim would be inappropriate. For the semi-generic practical impact score (daily activities and healthcare uptake questions), the correlation with the mental scale of the truly generic SF-36 instrument (involving mood and mental state) is satisfactory. In this light, the rather small effect size for disease activity on these partly generic daily activities and service uptake, only 0.173 SD, requires further probing. The strongest evidence for construct validity comes from the SD effect sizes for disease activity seen for hearing (0.795 SD) and ear symptoms (0.707 SD), both substantial, bordering on large, according to the most widely used system of anchor terms [17].

### Wider implications of factor structure for further development

By scientific criteria of parsimony, stability and adequacy on a mere 12 items, the 3-Factor solution was the most satisfactory one. The 2-F solution showed poor interpretability, but the issue remains of whether a 4-F solution could be shown to be preferable in other circumstances. Here, the 4-F was fairly similar to the 3-F; as frequently occurs, the addition of one factor came from a split between two roughly equal subsets of items loading on one existing factor -- the division between health service uptake and daily activities. A variety of circumstances could favour that split, but the most obvious possibility, relevant to international aggregation and international comparisons would be health culture and health systems. Where these differed, for example by the interposition of a financial barrier and so some patient selection by economic influences, a driver for healthcare uptake separate from impact on activities would be present, and so a factor split could be anticipated. The Serbian healthcare system has no major financial barrier of this nature.

### Strengths and limitations

In ENT where the level of psychometric expertise is not generally high, factor scores or other types of profile sub-score have sometimes been offered without due caution over the trade-off between general reliability and possible specific validity, the statistical strategy for extraction, the interpretation and the empirical adequacy of the scores used or offered [24]. As well as exemplifying a rigorous approach to decisions leading to sub-scores offered, our work has adhered to an overall statistical strategy, respecting parsimony and a priori constraints, and explicitly justifying decisions, such as the use of scaled values and number of factors extracted. Respect for empirical adequacy is seen in the test-re-test reliability and factor structure stability, and the report of effect sizes not merely of superficial ‘significance’, eg when claiming construct validity. We have addressed via item-scaling the fundamental issue of equal interval scaling, a prerequisite for using parametric statistics. We are aware that wider quantification of scaling’s benefits (the final recommendation of response-level scale values for precision and generality) awaits further work on a larger sample but have here justified that effort. Further documentation of three properties is still required; the wider impact of COM, given only slight effect of disease activity upon impact (healthcare uptake and daily activity limitations); generalisability to a wider range of patients including internationally; and responsiveness to change. Investment in such further work has also been justified by the staged questions addressed and the answers reached here.

## Conclusions

1. In its Serbian translation, COMQ-12 has good reliability, and the total score achieves conventional consistency.
2. The factor structure of the item content is retrieved with three factors as a basis for profile scoring, currently labelled as: ear problems, hearing problems and wider impact on daily activities plus healthcare uptake.
3. This structure is remarkably stable over 1 month, and scaled items retrieve it more satisfactorily than do unscaled; thus, metrical validity (the unit basis of scoring) has received attention in relation to a good factor solution.
4. Theoretical (construct) validity has been enhanced by an overall interpretable model relating the three factors and three external associated measures, embracing content and construct validities. Diagnosed cases of adult COM show a very large mean disease effect sizes of 2–3 SD relative to healthy controls. Subscore profiling with three factors is currently supportable for COMQ-12 in large samples, because each factor has at least three high-loading items. Although further items always add reliability, they do not appear to be needed to support this aim; thus standardizing the short-form item set seems not to have been premature.
5. Because response levels are influenced by item content, pending optimized item scaling on the larger phase 2 sample (to best serve profile scoring) the present scaled values offer a satisfactory way of getting more structure out of COMQ-12 datasets than alternative modes of scoring.

## Additional files

Additional file 1: Serbian version of Chronic Otitis Media Questionnaire 12 (COMQ-12) (DOCX 90 kb)

Additional file 2: Details of psychometric strategy. **a** Further notes on optimising precision and factor solution by scaling item response levels. **b** Factor loading patterns summarising factor solutions from 1st and 2nd visit data and their average (DOCX 23 kb)

## Abbreviations

(M)ANOVA: Multivariate Analysis Of Variance
CES: Chronic Ear Survey
CFA: Confirmatory Factor Analysis
COM: Chronic Otitis Media
COMOT-15: Chronic Otitis Media Outcome Test 15
COMQ-12: Chronic Otitis Media Questionnaire 12
EFA: Explanatory Factor Analysis
ES: Effect Size
HL: Hearing level (audiograms)
ICC: Intraclass Correlation Coefficient
PC: Principal Component
PROM: Patient-Reported Outcome Measure
QoL: Quality of Life
RMSEA: Root Mean Square Error Approximation
SD: Standard Deviation
SEM: Structural Equotation Modeling
SF-36: Short-Form 36 questionnaire
SRWs: Standardised Regression Weights

## References

[1] World Health Organization. Chronic suppurative otitis media. Burden of illness and management options 2004. Available at: http://www.who.int/pbd/publications/Chronicsuppurativeotitis_media.pdf.

[2] Sharon JD, Chole RA, Dornhoffer JL, Gluth MB (2016). Microbiology in chronic ear disease. The chronic ear.

[3] Alanis AJ (2005). Resistance to antibiotics: are we in the post-antibiotic era?. Arch Med Res.

[4] Harris AT, Mettias B, Lesser TH (2016). Pooled analysis of the evidence for open cavity, combined approach and reconstruction of the mastoid cavity in primary cholesteatoma surgery. J Laryngol Otol.

[5] Korsten-Meijer AG, Wit HP, Albers FW (2006). Evaluation of the relation between audiometric and psychometric measures of hearing after tympanoplasty. Eur Arch Otorhinolaryngol.

[6] Colletti V, Fiorino FG, Sittoni V (1987). Misculptured ossicle graft versus implants: long term results. Am J Otol.

[7] Black N (2013). Patient reported outcome measures could help transform healthcare. BMJ.

[8] Hallberg L, Hallberg U, Kramer SE (2008). Self-reported hearing difficulties, communication strategies and psychological well-being (quality of life) in patients with acquired hearing impairment. Disabil Rehabil.

[9] Nadol JB, Staecker H, Gliklich RE (2000). Outcomes assessment for chronic otitis media: the chronic ear survey. Laryngoscope.

[10] Baumann I, Kurpiers B, Plinkert PK, Praetorius M (2009). Development and validation of chronic Otitis media outcome test 15 (COMOT-15). Measurement of health-related quality of life in patients with chronic otitis media. HNO.

[11] Philips JS, Haggard M, Yung M (2014). A new health-related quality of life measure for active chronic otitis media (COMQ – 12): development and initial validation. Otol Neurotol.

[12] Van Dinther J, Droessaert V, Camp S, Vanspauwen R, Maryn Y, Zarowski A, Somers T, Offeciers E (2015). Validity and test-retest reliability of the Dutch version of the chronic otitis media questionnaire (COMQ – 12). J Int Adv Otol.

[13] Kosyakov SI, Minavnina JV, Philips JS, Yung MW (2017). International recognition of chronic Otitis media questionnaire 12. J Laryngol Otol.

[14] Bakir S, Kinis V, Bez Y (2012). Mental health and quality of life in patients with chronic otitis media. Eur Arch Otorhinolaringol.

[15] Ware JE, Sherbourne CD (1992). The MOS 36-item short-form health survey (SF-36). I. Conceptual framework and item selection. Med Care.

[16] Cumming G (2012). Understanding the new statistics: effect sizes, confidence intervals, and meta-analysis.

[17] Cohen J (1988). Statistical power analysis for the behavioral sciences.

[18] Kline RB (2015). Principles and practice of structural equation Modelling.

[19] Fayers MP, Machin D (2007). Quality of life. The assessment, analysis and interpretation of patient-reported outcomes.

[20] Milovanovic J, Filipovic SA, Marchisio P, Haggard MP, Zhang MF, Spencer H (2016). Precision-scored parental report questions and HL-scaled tympanometry as informative measures of hearing in otitis media 1: large-sample evidence on determinants and complementarity to pure-tone audiometry. Int J Pediatr Otorhinolaryngol.

[21] Kline P (1998). The new psychometrics: science, psychology and measurement.

[22] Phillips JS, Yung MW (2014). COMQ - 12 scores in adult patients without chronic middle ear disease. Clin Otolaryngol.

[23] Lutman ME, Brown EJ, Coles RRA (1987). Self-reported disability and handicap in the population in relation to pure-tone threshold, age, sex and type of hearing loss. Br J Audiol.

[24] Khalfa S, Dubal S, Veuillet E, Perez-Diaz F, Jouvent R, Collet L (2002). Psychometric normalization of a hyperacusis questionnaire. ORL J Otorhinolaryngol Relat Spec.

