# Supplementary material for: Psychometric characteristics of the chronic Otitis media questionnaire 12 (COMQ – 12): stability of factor structure and replicability shown by the Serbian version
Source: Health Qual Life Outcomes. 2017 Oct 23;15:207. doi: 10.1186/s12955-017-0782-x (PMC5651611; doi:10.1186/s12955-017-0782-x)
Supplement: Supplementary file 1 — Serbian version of Chronic Otitis Media Questionnaire 12 (COMQ-12) (DOCX 90 kb) [file 12955_2017_782_MOESM1_ESM.docx]

**Additional file 1. Serbian version of Chronic Otitis Media Questionnaire 12 (COMQ-12)**

| **Upitnik o hroničnom zapaljenju srednjeg uva-12 (COMQ-12):** |
| --- |
| Ova pitanja služe da saznamo koliko Vaši problemi sa uvom negativno utiču na Vas. Ne postoji mašina koja nam to može otkriti: samo Vi nam to možete reći. Očekujemo da će nam rezultati ovog upitnika pomoći da razumemo koji je od Vaših simptoma koji se odnose na uvo najznačajniji za Vas. Saznanje o tome će nam pomoći da poboljšamo načine na koje brinemo o pacijentima sa problemima sa uvom. |
| Molimo Vas da odgovorite na pitanja navedena ispod tako što ćete svako postavljeno pitanje pažljivo razmotriti i potom zaokružiti odgovarajući broj; svaki broj odgovara određenom opisu. Ne postoje tačni i netačni odgovori, ali Vas molimo da pažljivo razmislite o svakom pitanju pre zaokruživanja odgovarajućeg broja. Molimo Vas da razmotrite svaki problem u odnosu na to kakav je bio u poslednjih 6 meseci. |
| **PRIMER:** |
| Za sledeća pitanje Vas molimo da označite koliko često upražnjavate ovu aktivnost koristeći skalu navedenu ispod i zaokruživanjem odgovarajućeg broja: |
| 0 Nikada |
| 1 Najmanje jednom u svaka 3 meseca |
| 2 Najmanje jednom svakog meseca |
| 3 Najmanje jednom nedeljno |
| 4 U većini dana u nedelji |
| 5 Sve vreme |
| **Koliko često jedete tost za doručak?** 0 1 2 3 4 5 |
|  |
| Osoba koja ovako odgovara saopštava da obično jede tost, ali ne uvek. |
| Ukoliko imate bilo kakvih problema sa odgovaranjem na pitanja, molimo Vas da zamolite člana kliničkog osoblja da Vam pomogne. |
| Hvala Vam. |

| Za sledeća pitanja Vas molimo da označite *koliko teško* razni elementi koji su opisani utiču na Vas, koristeći skalu navedenu ispod i zaokruživanjem odgovarajućeg broja: |
| --- |
| 0 Uopšte mi ne smeta |
| 1 Mala nelagodnost |
| 2 Umerena nelagodnost |
| 3 Velika nelagodnost, ali mogu da se nosim s njom |
| 4 Velika nelagodnost i teško mi je da se nosim s njom |
| 5 Najgora stvar koja je ikada uticala na moj život |
| **Težina simptoma:**   \| 1. Curenje ili dreniranje iz uva \| 0 \| 1 \| 2 \| 3 \| 4 \| 5 \| \| --- \| --- \| --- \| --- \| --- \| --- \| --- \| \| 2. Prisustvo neprijatnog mirisa iz uva \| 0 \| 1 \| 2 \| 3 \| 4 \| 5 \| \| 3. Problemi sa sluhom kod kuće, npr. koji zahtevaju pojačavanje zvuka na televizoru ili radiju \| 0 \| 1 \| 2 \| 3 \| 4 \| 5 \| \| 4. Problemi sa sluhom kada razgovarate sa ljudima u grupama ili kada ste u bučnom okruženju \| 0 \| 1 \| 2 \| 3 \| 4 \| 5 \| \| 5. Nelagodnost u i/ili u okolini uva \| 0 \| 1 \| 2 \| 3 \| 4 \| 5 \| \| 6. Vrtoglavica ili osećaj „gubitka ravnoteže” \| 0 \| 1 \| 2 \| 3 \| 4 \| 5 \| \| 7. Zujanje ili šumovi u uvu \| 0 \| 1 \| 2 \| 3 \| 4 \| 5 \| |

| Za sledeća pitanja Vas molimo da označite *koliko često* razni elementi koji su opisani utiču na Vas, korišćenjem skale ispod i zaokruživanjem odgovarajućeg broja: |
| --- |
| 0 Ređe od jednom u svakih 6 meseci |
| 1 Najmanje jednom u svakih 6 meseci |
| 2 Najmanje jednom u svaka 3 meseca |
| 3 Najmanje jednom svakog meseca |
| 4 Najmanje jednom nedeljno |
| 5 U većini dana u nedelji |
| **Uticaj na način života i posao:** |
| Koliko često NISTE bili u mogućnosti da:   \| 8. Obavljate Vaše uobičajene svakodnevne aktivnosti kod kuće ili na poslu? \| 0 \| 1 \| 2 \| 3 \| 4 \| 5 \| \| --- \| --- \| --- \| --- \| --- \| --- \| --- \| \| 9. Da se perete, tuširate ili kupate kako biste voleli? To jest, koliko često ste bili uplašeni da bi ove aktivnosti mogle izazvati infekciju uva? \| 0 \| 1 \| 2 \| 3 \| 4 \| 5 \| |
| **Uticaj na zdravstvenu službu:**   \| 10. Koliko često ste išli kod Vašeg lekara opšte prakse zbog Vaših problema sa uvom? \| 0 \| 1 \| 2 \| 3 \| 4 \| 5 \| \| --- \| --- \| --- \| --- \| --- \| --- \| --- \| \| 11. Koliko često morate da uzimate lekove (uključujući i kapi za uši) za Vaš problem sa uvom? \| 0 \| 1 \| 2 \| 3 \| 4 \| 5 \| |
| Za sledeće pitanje Vas molimo da označite koliko su stvari loše, na skali od „0” do „5”. |
| „0” označava nimalo, a „5” označava najgore što ste ikada mogli da zamislite: |
| **Uopšteno:**   \| 12. Do kog stepena Vaši problemi sa uvom negativno utiču na Vaše raspoloženje? \| 0 \| 1 \| 2 \| 3 \| 4 \| 5 \| \| --- \| --- \| --- \| --- \| --- \| --- \| --- \| |
| Molimo Vas da proverite da li ste odgovorili na svako pitanje i potražite pomoć ukoliko imate poteškoća.  Hvala Vam puno što ste učestvovali. |
