# Supplementary material for: Psychometric characteristics of the chronic Otitis media questionnaire 12 (COMQ – 12): stability of factor structure and replicability shown by the Serbian version
Source: Health Qual Life Outcomes. 2017 Oct 23;15:207. doi: 10.1186/s12955-017-0782-x (PMC5651611; doi:10.1186/s12955-017-0782-x)
Supplement: Supplementary file 2 — Details of psychometric strategy. a Further notes on optimising precision and factor solution by scaling item response levels. b Factor loading patterns summarising factor solutions from 1st and 2nd visit data and their average (DOCX 23 kb) [file 12955_2017_782_MOESM2_ESM.docx]

**Additional file 2. Details of psychometric strategy**

**Additional file 2a. Further notes on optimising precision and factor solution by scaling item response levels**

Unless the number of data points is very large (here it is sufficient at 120), the decision to use scaled values may be uncertain or marginal. This is because scaling with only a small sample can introduce some roughness as seen for example where values are shared between adjacent response levels (eg in Table 2, levels 2 and 3 for item 1). Roughness can result from necessity of pooling categories due to rarity of use of a particular level (as in this example), which having a large N can eventually overcome. Such roughness, due to imprecision of estimation in an only medium-sized sample, can nevertheless be more than offset by the elimination of the systematic scale distortion introduced by the usual incorrect practice of taking response data-entry codes 1, 2, 3 etc at face-value, as is illustrated for the response levels 4 and 5 in the main text, for choice of factor solution. Table 2 shows that only three out of twelve items (4, 6 and 12) achieve substantial differentiation in the total between the response levels of ‘4’ and ‘5’; for the other 9, the differentiation is more slight here than for the other steps between response levels, and even flattened or reversed. Further violations of the face-value integer response level coding, which it is inefficient to ignore are seen. The extreme dichotomisation of item 9 is necessary because of extreme skew of the distribution of response levels, and a plateau at the lower end, as in item 2 and to some extent in items 4, 5, and 7. Adopting the scaled values for response levels turns this evident difference between items to efficient use, as patterning replaces roughness with increasing sample size.

The principle of attending to equal interval measurement as assumed by totalling and other procedures in score derivation is incontestable. More debatable, and demanding impartial follow-up evaluation on a large sample is whether for a given topic factor structure, a given participant sample, the response scale presented, and the sample size available to narrow the errors on the estimates for rarer levels of response, the advantages are material enough to be worth pursuing. Having pursued an optimising approach for a validation criterion measure, the measure from the instrument offered for field use may in fact be simplified once more (eg raw total), having been validated by high correlation with this optimum. Other evaluations than the present one of improving the factor structure are required. Previous work with a large sample^20^, showed improvement of correlations and improvement of linearity by reduction of ceilng effects. These considerations play out in the next additional file 2b and Table 3 concerning the choice of factor solution. We envisage re-scaling, but with the high-loading items scaled to factors, rather than to the principal component as preliminary total, this will become more efficient when a larger sample reduces the standard errors on the estimates (which provide the scaled values) for the more rare (extreme) repsonses.

**Additional file 2b. Factor loading patterns summarising factor solutions from 1^st^ and 2^nd^ visit data and their average**

|  | **V1 3-FAC** | | | **V2 3-FAC** | | | **V12 average 3-FAC** | | | **V12 average 4-FAC** | | | |
| --- | --- | --- | --- | --- | --- | --- | --- | --- | --- | --- | --- | --- | --- |
| **Q** | **FAC1** | **FAC2** | **FAC3** | **FAC1** | **FAC2** | **FAC3** | **FAC1** | **FAC2** | **FAC3** | **FAC1** | **FAC2** | **FAC3** | **FAC4** |
| **1** | .199 | .242 | **.669** | .251 | .168 | **.732** | .222 | .221 | **.705** | *.319* | .250 | **.686** | -.021 |
| **2** | .151 | -.012 | **.764** | .081 | .146 | **.791** | .120 | .073 | **.791** | .191 | .079 | **.781** | -.013 |
| **3** | .147 | **.874** | .093 | .214 | **.898** | .008 | .185 | **.884** | .059 | .202 | **.884** | .055 | .185 |
| **4** | .099 | **.841** | .216 | .087 | **.838** | .212 | .085 | **.833** | .230 | .160 | **.868** | .226 | .056 |
| **5** | -.008 | **.536** | *.528* | .109 | **.622** | *.458* | .062 | **.600** | *.482* | -.096 | *.454* | **.506** | *.489* |
| **6** | **.629** | *.377* | -.080 | **.663** | .229 | -.125 | **.650** | *.342* | -.128 | *.364* | .135 | -.131 | **.759** |
| **7** | .273 | **.604** | .068 | *.337* | **.492** | .146 | *.310* | **.575** | .093 | .137 | *.439* | .101 | **.529** |
| **8** | **.623** | .240 | *.343* | **.714** | .267 | .212 | **.668** | .271 | .281 | **.669** | .249 | .244 | .233 |
| **9** | **.585** | -.034 | *.485* | **.661** | -.059 | *.377* | **.631** | -.040 | **.429** | **.485** | -.174 | *.409* | *.468* |
| **10** | **.841** | .119 | .250 | **.795** | .142 | .243 | **.826** | .126 | .249 | **.873** | .139 | .196 | .149 |
| **11** | **.850** | .107 | .214 | **.808** | .183 | .224 | **.833** | .141 | .228 | **.874** | .150 | .175 | .164 |
| **12** | *.307* | .240 | **.668** | *.481* | .259 | **.553** | *.398* | .269 | **.608** | .228 | .109 | **.609** | *.537* |

This additional file Table provides the evidence for the decision to average data to produce the most reliable and generalisable factor analytic solution from the 120 data points; this in turn underpins the decision not to extract a fourth factor. Rows are the questionnaire items in their standard numbering, 1-12 identifiable from Table 2 for interpretation purposes. The four upper headings to the four fields refer to the data source for the analysis (and number of factors extracted) to provide a scoring formula, which can be imposed on any set of data: the very same, or another visit or other participants, or another sample in another country, to calculate individuals’ scores. The high loadings (>0.45) identifying factors are in bold, and the cross-loadings > 0.30 are in italic. The first issue is the similarity of the first two 3-column fields, which can already be seen in the bold patterning. More precisely, secondary correlations between visits across items’ loading values (themselves a type of correlation) exploit the most powerful index of agreement for any type of data, it being irrelevant that primary correlations across individuals also underpin factor analysis. The visit 1-to-visit 2 (first field to third field), secondary r-values between the respective 12 pairs of loadings for the 1^st^, 2^nd^ and 3^rd^ factors, column 1 with column 3 etc, are 0.970, 0.961, and 0.967. This establishes the remarkably high stability (replication) of the factor solution. It also justifies use of the third field, the visit-averaged data (ie averaging the scaling coefficients appropriate to the response levels in the two pieces of data at the item level).

Visit-averaged data give the most powerful and generalizable data reduction, and the appropriate comparator for assessing the 4^th^ field, the potential 4-factor solution. Correlations are not so appropriate for differing numbers of factors extracted, because at least two factors from a 4-F solution can have no comparable counterpart in the corresponding 3-F. This said, lowest-numbered factors are often hardly altered by extracting a further factor (confirmed for the 7^th^ with 10^th^ columns of the field), and others, or may be only slightly altered (8^th^ with 11^th^). Here it is indeed the 3^rd^ factor which fissures to provide the fine detail in 3^rd^ and 4^th^ factors of 4-F; but these factors also attract one high-loading item each from Factor 1 (Q6) and Factor 2 (Q7), not strongly present in the 3^rd^ factor of 3-F. These two migrants are the wild-card items for tinnitus and dizziness, each retaining one of the cross-loadings (italic) with its 3-F origin. They thus contribute, on splitting to 4-F, to the quite high total of seven strong cross-loadings in 4-F compared to only three in the 3-F solution. This total argues against 4-F. In migrating for 4-F the wild card items increase rather than reduce cross-loading, and seem to drive the fourth factor more with their own (anticipated) problematic nature than any inherent similarity. Additionally ‘discomfort’ is a cross loader, but it is not entirely surprising that an infection severe enough to cause discomfort would also cause hearing loss. The balance of considerations favouring the 3-F over the 4-F solution is set out in the main text with recourse to Table 3.

If we accept a loading of 0.45 or above as ‘high’, we are left with the following as highest loadings identifying the factors:

Factor 1 : impact on daily activities and healthcare uptake (Q11 medicines, Q10 GP visits, Q8 activity restriction, Q6 dizziness, Q9 limiting water exposure).

Factor 2: hearing problems (Q 3 hearing at home, Q4 hearing in noise , Q5 ear discomfort, Q7 tinnitus).

Factor 3: ear problems (Q1 discharging ear, Q2 smelly ear, Q12 ‘down’ - overall effect on QoL, Q5 – the main cross-loading in the grounded SEM in Figure 1 – ‘discomfort’).
